# SPARC expression in gastric cancer predicts poor prognosis: Results from a clinical cohort, pooled analysis and GSEA assay

**DOI:** 10.18632/oncotarget.12191

**Published:** 2016-09-22

**Authors:** Zhi Li, Ao-Di Li, Lu Xu, De-Wei Bai, Ke-Zuo Hou, Hua-Chuan Zheng, Xiu-Juan Qu, Yun-Peng Liu

**Affiliations:** ^1^ Department of Medical Oncology, The First Hospital, China Medical University, Shenyang, Liaoning Province, China; ^2^ Department of Cell Biological Treatment Ward, Dalian Centre Hospital, Dalian, Liaoning Province, China; ^3^ Life Science Institute of Jinzhou Medical University, Jinzhou, Liaoning Province, China; ^4^ Key Laboratory of Anticancer Drugs and Biotherapy of Liaoning Province, Shenyang, Liaoning, China

**Keywords:** SPARC, gastric cancer, prognosis, immunohistochemistry, GSEA

## Abstract

**Background:**

The prognostic role of Secreted Protein Acidic and Rich in Cysteine (SPARC) in gastric cancer (GC) remains controversial. We investigated the clinical significance, the survival relevance, and potential function of SPARC in GC with resected samples, online gene set GSE62254, and cell line SGC7901.

**Results:**

High immunostaining of SPARC significantly correlated with tumor differentiation (*P* = 0.004), and independently predicted shorter overall survival (OS) (HR = 1.446, *P* = 0.022), based on the current IHC evaluation. The accuracy of the results was further validated with 1000 times bootstrapping and the time-dependent receiver-operating characteristics (ROC) curves. The meta-analysis (pooled HR = 1.60, 95% CI: 1.01−2.53) confirmed SPARC as the predictor for reduced OS in GC. Moreover, the association between enhanced SPARC expression and Adriamycin (Adr) sensitivity was revealed by GSEA, and then confirmed by comparative cellular experiments, such as the protein level analysis of SGC7901and SGC7901/Adr cell line.

**Materials and Methods:**

Immunohistochemistry (IHC) method was used to detect SPARC expression in 137 GC cases. Meta-analysis was performed based on 5 studies published in English on PubMed up to March 2016. GSEA was performed using online data set GSE62254 and GC-related functional gene sets derived from molecular signatures database (MSigDB). Western Blot was carried out to compare protein-level differences between gastric carcinoma SGC7901 cell line and Adr resistant SGC7901/Adr cell line. MTT assay was done to confirm the induction of SPARC on Adr sensitivity

**Conclusions:**

Increased SPARC expression in GC led to a worse clinical outcome of patients and might induce Adr sensitivity of GC cells.

## INTRODUCTION

Despite the therapy development in recent years, gastric cancer (GC) remains one of the leading causes of cancer death in the world [1], partly due to the absence of effective therapeutic targets and prognostic markers. Dozens of molecules predictive for cancer progression and prognosis are discovered each year, whereas seldom is applied in clinical settings because of limited consistency between biological and clinical data and low repeatability of multiple studies with different samples or methods [2].

Yet remarkably, the unique matricellular glycoprote Secreted Protein Acidic and Rich in Cysteine (SPARC) is gaining increasing attention, either for its extensive biological effect on tumor development, invasion, metastasis, angiogenesis and inflammation by mediating cell-microenvironment interaction [3], or for the predictive potential for the efficacy of nanoparticle albumin-bound (NAB) chemotherapy drugs via inducing drug accumulation [4]. Biologically, SPARC was found to promote cancer development in some tumors with highly metastatic characteristics, such as breast cancer and melanoma, but act as a tumor suppressor in some other cancer types [5, 6]. Clinically, SPARC has been uncovered as the potential prognostic marker in several cancer types [7–9].

In GC, the function of SPARC is still controversial. Some clinical studies identified SPARC as a predictor of worse prognosis for GC [10–12], whereas others found insignificant/inverse results using comparable methods [13, 14]. So far, there was only one meta-analysis for the prognostic value of SPARC in GC. However, one study showing maximum weight in the fix-effect meta-analysis extracted data based on the non-SPARC-specific survival curve [15]. Meanwhile, some laboratory studies on cell lines and animal models showed that SPARC could attenuate the angiogenesis but inhibit the proliferation of tumor [16–18], whereas others indicated SPARC to promote cancer development, invasion and metastasis [19]. Therefore, the overall role of SPARC in GC remains to be unraveled either biologically, clinically or systematically.

The current study revealed the clinicopathological significance of SPARC in GC based on 4 distinct lines of investigation. SPARC was the potential predictor for the progression and prognosis of GC, as demonstrated by the IHC evaluation of our own cohort of 137 GC cases, externally validated by a meta-analysis of 5 English-published studies, and further supported by the bioinformatical assay of online dataset, and our own cellular experiments.

## RESULTS

### Patient characteristics

As shown in Table 1, the cohort contained 137GC patients (103 men, 34 women), and the median age at surgery was 60 years old. D2 lymph node dissection was performed in 131 cases. Based on the criteria of American Joint Committee on Cancer (AJCC) (7th edition), the majority (78.1%) of patients had advanced TNM stage, and unexpected metastases were found in 4 cases (2.9%) during the surgery and postoperative pathology examination. Patients with stage I–II did not receive adjuvant chemotherapy. Patients with stage III received 5-fluorouracil (5-Fu) based adjuvant chemotherapy. Patients with stage IV received salvage chemotherapy. The median survival duration was 1028 days (range: 8–1859 days).

**Table 1:** Characteristics of the gastric cancer study cohort

Characteristics	Number of patients (%)
Age (years)	
Median (range)	60.0 (38.0–78.0)
Gender	
Female	34 (24.8)
Male	103 (75.2)
Surgery D2	
Yes	131 (95.6)
No	6 (4.4)
Location	
Fundus & cardia	8 (5.8)
Body	55 (40.1)
Antrum & pylous	74 (54.0)
Differentiation	
Well & moderate	62 (45.3)
Poor & mixed	75 (54.7)
Lauren type	
Intestinal	59 (43.1)
Diffused	78 (56.9)
T stage	
T1–T3	29 (21.2)
T4	108 (78.8)
N stage	
N0	31 (22.6)
N1–N3	106 (77.4)
M stage	
M0	133 (97.1)
M1	4 (2.9)
TNM Stage	
I & II	30 (21.9)
III & IV	107 (78.1)

### SPARC expression and its association with clinicopathological variables

**Figure 1:**
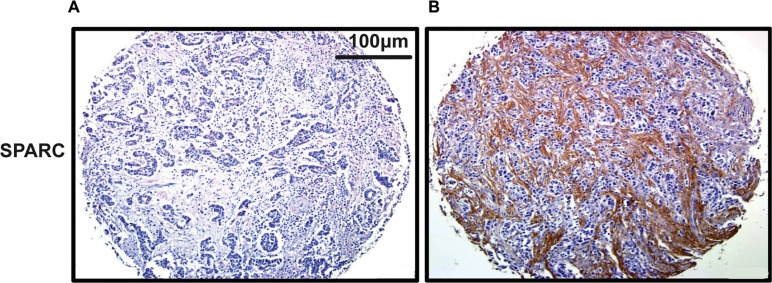
Representative staining of SPARC in gastric cancer tissue by IHC (200×) (**A**) No staining of SPARC in cancer tissue; (**B**) Positive SPARC staining in the surrounding desmoplastic stroma.

SPARC staining was weak in cancer cells, while exhibited a relatively strong signal in the cytoplasm of surrounding stromal cells. Among the 137 cancer specimens in the current study, 84 (61.3%) demonstrated high immunoactivity of SPARC (Figure 1). The association of SPARC expression with clinicopathological characteristics of the cohort is shown in Table 2. Enhanced SPARC staining was significantly correlated with differentiation (*P* = 0.01) and Lauren type (*P* = 0.02) of cancer, but not with the other clinicopathological variables, such as the age and gender of patients, and the surgical procedure, location, invasion depth, lymph node involvement, and distant metastasis of cancers.

**Table 2:** Association of SPARC expression in cancer stroma with clinicopathologic parameters

Characteristics	Low expression of SPARC, *n* (%)	High expression of SPARC, *n* (%)	*P*-value
Age (years)			0.56
Mean +/− SD	60.2 +/− 10.7	59.1 +/− 10.2	
Gender			0.73
Female	14 (26.4)	20 (23.8)	
Male	39 (73.6)	64 (76.2)	
Surgery D2			0.88
Yes	50 (94.3)	81 (96.4)	
No	3 (5.7)	3 (3.6)	
Location			0.06
Fundus & cardia	4 (7.5)	4 (4.8)	
Body	16 (30.2)	39 (46.4)	
Antrum & pylous	33 (62.3)	41 (48.8)	
Differentiation			0.01
Well & moderate	32 (60.4)	30 (35.7)	
Poor & mixed	21 (39.6)	54 (64.3)	
Lauren type			0.02
Intestinal	30 (56.6)	29 (34.5)	
Diffused	23 (43.4)	55 (65.5)	
T stage			0.46
T1–T3	9 (17.0)	20 (23.8)	
T4	44 (83.0)	64 (76.2)	
N stage			0.83
N0	13 (24.5)	18 (21.4)	
N1–N3	40 (75.5)	66 (78.6)	
M stage			0.96
M0	52 (98.1)	81 (96.4)	
M1	1 (1.9)	3 (3.6)	

### Influence of SPARC expression on survival

**Figure 2:**
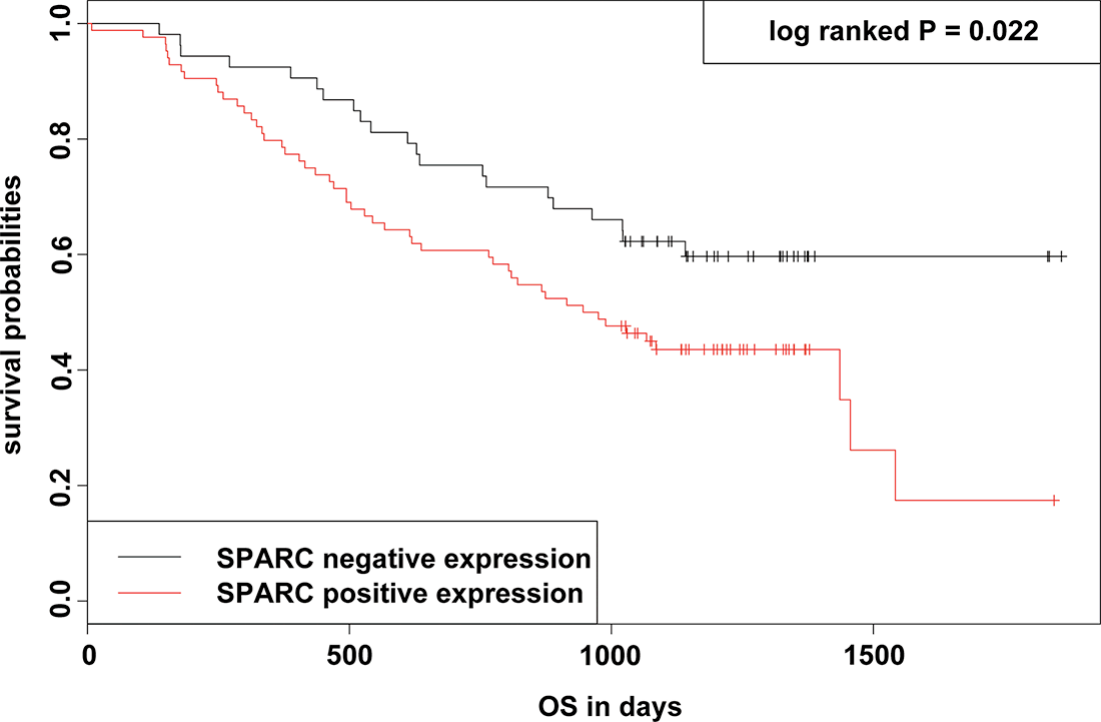
KM survival curve and log-rank test for patients classified as showing either positive or negative SPARC expression in GC Patients with SPARC high expression exhibited a significant worse survival than those with SPARC low expression (*P* = 0.022; log-rank test).

**Figure 3:**
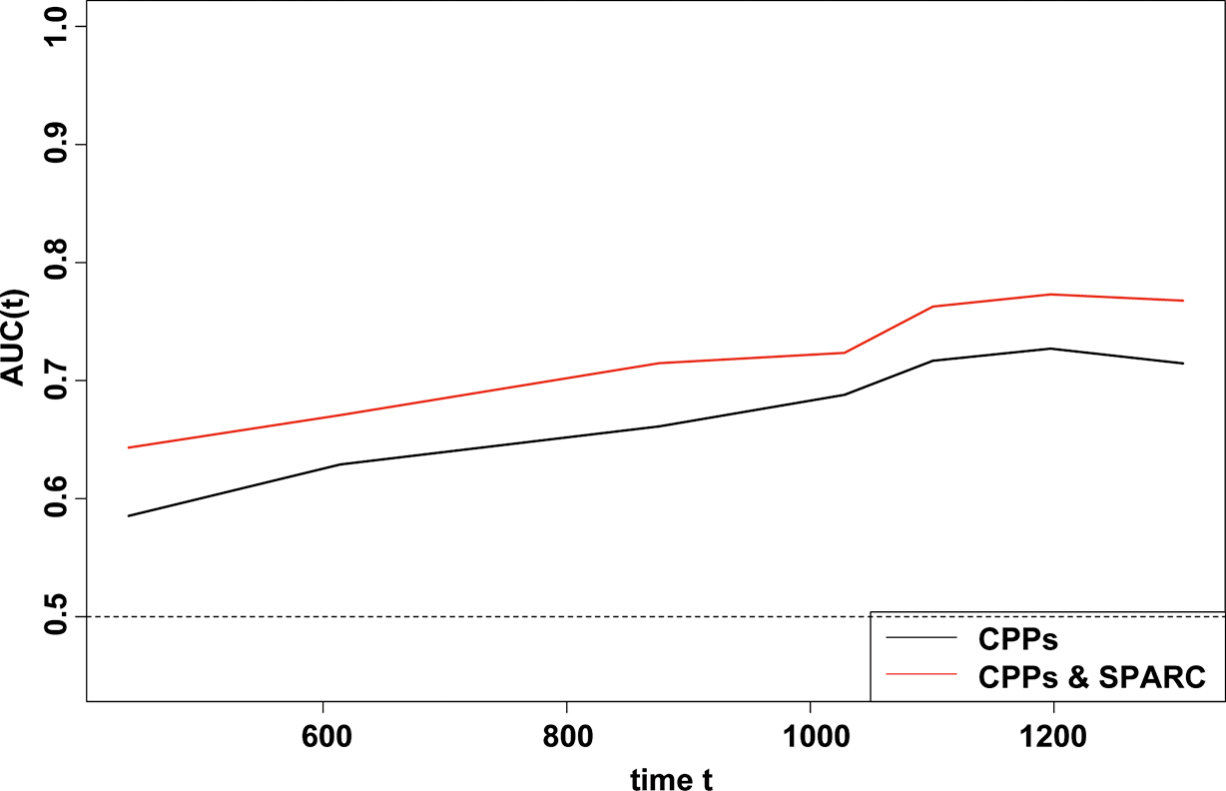
Time-dependent ROC analyses for the CPPs, and the combination of SPARC and CPPs The inclusion of the SPARC expression score in the model improved the predictive ability slightly.

In a Kaplan-Meier (KM) analysis, SPARC expression was significantly related with overall survival (OS), and patients with high SPARC expression usually demonstrated shorter OS (log-rank *P* = 0.022) (Figure 2). Univariate analysis using COX proportional hazard (PH) models showed that advanced T stage, presence of lymph node metastasis, and high SPARC expression significantly predicted reduced OS (*P* = 0.007, 0.001, and 0.024, respectively) (Table 3). In the multivariate COX PH analysis, SPARC expression (Hazard ratio (HR) = 1.835, *P* = 0.022), surgical procedure (HR = 0.296, *P* = 0.024), T stage (HR = 3.032, *P* = 0.005), and N-stage (HR = 3.866, *P* = 0.002) were revealed independent indicators for OS, and the prognostic model for GC were further validated using 1000 times bootstrapping (Table 3). Moreover, the predictive ability of the prognostic model was slightly improved by the inclusion of SPARC level (Figure 3), as demonstrated by the increase of the resulting area under the curve (AUC) value from 0.798 to 0.811 at the 5th year of follow up.

**Table 3:** Univariate and multivariate analyses of overall survival according to clinicopathological parameters and SPARC levels with 1000 bootstraping

Characteristics	No		Uni–variant analysis			Multi–variant analysis			
	Patients	Events	HR	95% CI	*P*–value	HR	95% CI	*P*–value	Bootstrapping 95% CI
Age (years)	137	71	1.011	0.988–1.034	0.368				
Gender			1.124	0.635–1.988	0.689				
Female	34	15							
Male	103	56							
Surgery D2			0.508	0.185–1.396	0.189	0.296	0.103–0.852	0.024	0.076–0.847
Yes	131	67							
No	6	4							
Location			0.750	0.516–1.089	0.130				
Fundus & cardia	8	6							
Body	55	31							
Antrum & pylous	74	34							
Differentiation			1.047	0.655–1.675	0.847				
Well & moderate	62	33							
Poor & mixed	75	38							
Lauren type			1.627	0.998–2.650	0.051				
Intestinal	59	25							
Diffused	78	46							
T stage			2.769	1.313–5.837	0.007	3.032	1.394–6.594	0.005	1.608–8.174
T1–T3	29	8							
T4	108	63							
N stage			3.917	1.692–9.067	0.001	3.866	1.664–8.982	0.002	1.865–10.979
N0	31	6							
N1–N3	106	65							
M stage			1.409	0.431–4.609	0.571				
M0	133	68							
M1	4	3							
SPARC expression			1.798	1.080–2.995	0.024	1.835	1.093–3.083	0.022	1.179–3.401
Negative	53	21							
Positive	84	50							

Additionally, survival analysis with online GC dataset (GSE62254) also showed that SPARC expression was significantly related with OS, and patients with high SPARC expression demonstrated shorter OS (log-rank *P* = 0.004) and disease free survival (DFS) (log-rank *P* < 0.001) (Supplementary Figure S3).

### Meta-analysis for the prognostic role of SPARC in GC as an external validation

**Figure 4:**
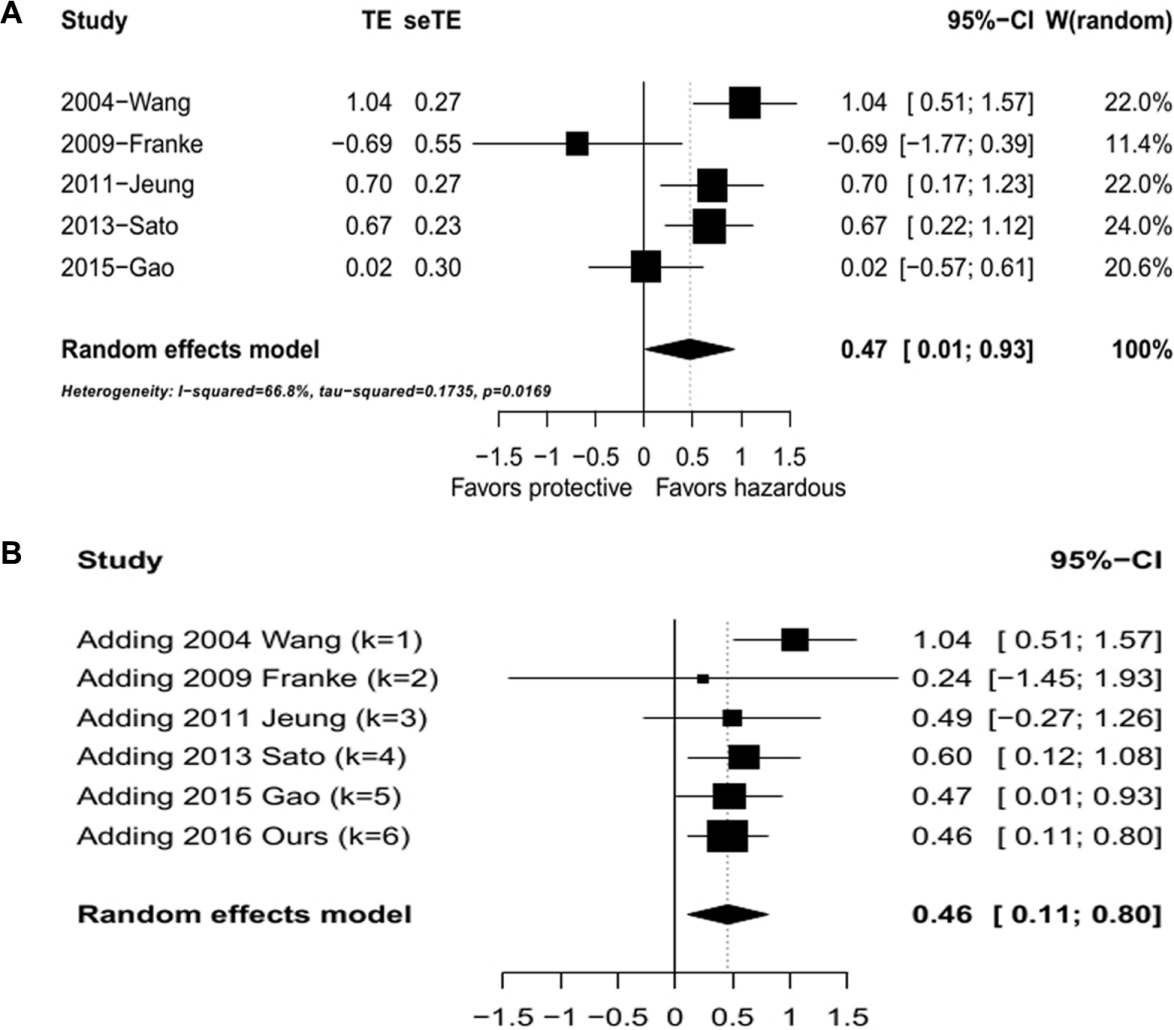
Meta analysis of overall survival against SPARC levels (**A**) Hazard ratio plot of overall survival against SPARC levels. (**B**) Hazard ratio plot of studies of overall survival against SPARC expression with cumulative meta analysis.

A PRISMA flow diagram of the literature search and selection is presented in Supplementary Figure S1. There were totally 5 studies including 566 patients (ranging from 43 to 227 patients per study) were included in the current meta-analysis and the main characteristics of the eligible studies were summarized in Supplementary Table S1. The pooled HR was calculated to unravel the association of SPARC expression with OS using the methods described in the Supplementary File S1. As the test for heterogeneity was significant (*I*^2^ = 66.8%, *P* = 0.0169), a random-effect model was used. High SPARC expression was highly correlated with reduced OS (pooled HR = 1.60, 95% confidence interval (CI): 1.01–2.53, transformed from lnHR and its 95% CI; Figure 4A), and the further chronologically cumulative meta-analysis demonstrated that our current study enhanced the combining effect favoring the prognostic role of SPARC in GC (pooled HR = 1.59, 95% CI: 1.12–2.23, transformed from lnHR and its 95% CI; Figure 4B). In addition, there was no publication bias of the eligible studies demonstrated by the funnel plot and an Egger's test (*P* = 0.1404, Supplementary Figure S2).

### Gene set enrichment analysis (GSEA) analysis for SPARC expression in GC

**Figure 5:**
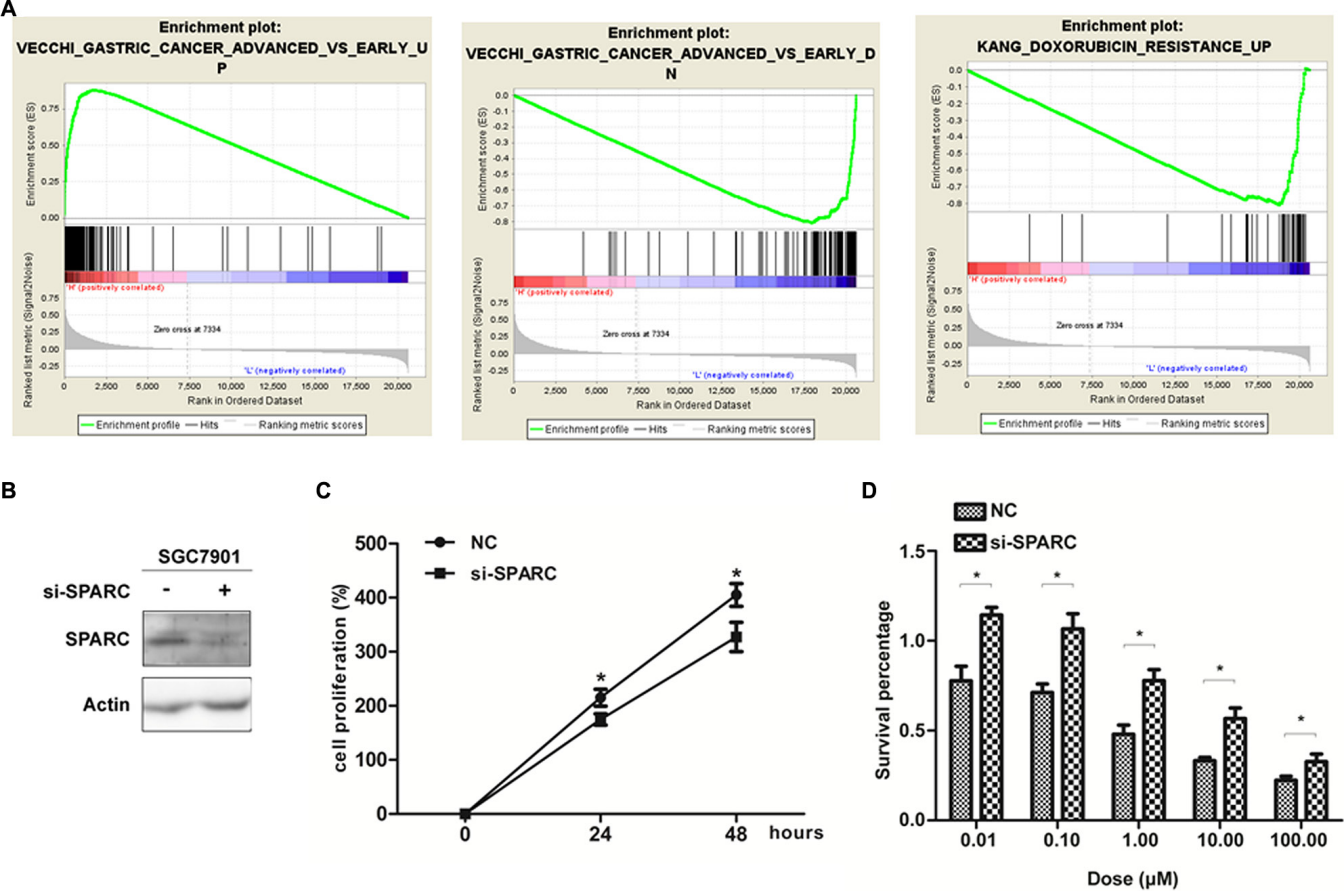
SPARC expression and GC proliferation as well as drug sensitivity properties (**A**) The GSEA results showing the correlation of SPARC levels and GC related gene sets in MSigDB. gene set “gastric cancer advanced vs early up (Vecchi)” was enriched in SPARC high expression phonotype (left), gene sets “gastric cancer advanced vs early down (Vecchi)” (middle), and “doxorubicin resistance up (Kang)” (right), were enriched in SPARC low expression phonotype. (**B**) Western-blot analysis for the protein level of SPARC in SGC7901 transfected with SPARC siRNA for 24 hours. (**C**) MTT assay showing SPARC knock-down inhibited SGC7901proliferation compared with control cells. (**D**) MTT assay showing the Adr-sensitivity of SGC7901 with SPARC siRNA transfection for 24 hours. Each data point represents the mean ± SD of three independent experiments. (*P < 0.05).

To investigate biologic characteristics shared by the different SPARC expression levels, we performed GSEA assay, a robust computational method that determines whether an a-priori defined set of genes shows statistically significant, concordant differences between both groups. The most significant pathways for both up- and down-regulated gene sets in the significance order (size of FWER *P* values) are listed in the Supplementary Table S2 and Figure 5.Three pathways, including “VECCHI_GASTRIC_CANCER_ADVANCED_VS_EARLY_UP”, “KIM_LRRC3B_TARGETS”,and “NOJIMA_SFRP2_TARGETS_UP”, were significant in SPARC high expression phenotype, and three pathways, including “VECCHI_GASTRIC_CANCER_ADVANCED_VS_EARLY_DN”, “KANG_DOXORUBICIN_RESISTANCE_UP”, and “KANG_FLUOROURACIL_RESISTANCE_DN”, were significant in SPARC low expression phenotype. The results indicated SPARC high expression induced GC progression and sensitivity to chemotherapy treatment such as adriamycin (Adr) and 5-Fu.

### SPARC increase Adr sensitivity demonstrated by cellular experiments

Our previous data showed a high expression level of SPARC in SGC7901 GC cell line [20]. SPARC was decreased in SGC7901/Adr cell line, comparing with the parental cell line SGC7901 (Supplementary Figure S4). This result is consistent with the findings in our microarray data, which compared the mRNA expression levels between SGC7901/Adr and SGC7901 with Affymetrix HGU133 Plus2 chips (data not shown). In order to confirm the induction of SPARC on Adr sensitivity, we did MTT experiment using siRNA to knock down SPARC expression in GC cell line SGC7901. The results indicated that Adr resistance was increased in SGC7901 cells with reduced SPARC expression.

## DISCUSSION

The current study firstly investigated the clinicopathological significance and the potential function of SPARC in GC simultaneously with multiple methods including IHC assay, meta-analysis, bioinformatical assay, and cellular experiments. The IHC staining of our own 137 GC specimens revealed that high SPARC expression significantly correlated with poorer differentiation and diffuse type of cancer, and independently predicted shorter OS. The meta-analysis of 5 available English publications on PubMed further validated SPARC to be an independent predictor for worse prognosis of GC patients. Using online data GSE62254, survival analysis confirmed the prognostic value of SPARC in GC, and GSEA results demonstrated the association of high SPARC expression with GC progression and sensitivity to treatment of Adr and 5-Fu. Further cellular experiments confirmed the induction of Adr sensitivity by SPARC in GC.

Similar to our results, the close association between SPARC expression and worse differentiation was previously observed in ovarian and prostate cancer, and was supposed to be due to the modulation of SPARC on the cell-matrix interactions [21, 22]. These results might be explained by the following clues: GC cell differentiation was accompanied with decreased expression of WNT signaling, and the activated WNT signal could increase the expression of SPARC [23, 24]. The correlation between WNT/SPARC and GC differentiation would be an intriguing topic for further investigation. It was also noticed that SPARC expression was either statistically meaningless for the prediction of cancer differentiation or significantly related with better cancer differentiation in some previous studies in GC [10, 12]. We supposed that the discrepant conclusions from the above studies might attribute to the heterogeneity of GC.

The previous clinical studies could not get a consistent conclusion about the association of SPARC with patient survival. We currently demonstrated SPARC to be the independent predictor for reduced OS in GC. Combining our current study and the previous report, the prognostic role of SPARC on GC is made robust based on the following several lines of evidence: 1) The current immunohistochemistry (IHC) assay was performed on a cohort of more than one hundred cases, and validated by a strict bootstrapping procedure; 2) The cumulative meta-analysis combining with our current IHC assay reached a consistent conclusion with a more narrow 95% CI, comparing with the non-cumulative analysis without our study; 3) Both survival analysis and GSEA results using an online dataset reached the coincident conclusion; and 4) Some previous biological experiments declared that down-regulation of SPARC could induce the growth and invasion recession of GC cells, and might explain the poor patient prognosis mediated by SPARC. All these suggested the potential value of this marker for clinical prognostic prediction in GC.

The distribution and the related function of SPARC in the stroma and cancer cells is an intriguing topic. We and some other scientists have demonstrated that SPARC in cancer cells could regulate the apoptosis, prohibit the angiogenesis and promote the invasion and the proliferation of tumors [18–20, 25]. In cancer stroma, SPARC regulates extracellular matrix (ECM) assembly, exhibits anti-adhesive function, and promotes emigration and invasion [3]. In our current meta-analysis, the expression of SPARC in GC, no matter the location, is the independent predictor for poor prognosis of patients, and the conclusion is well supported by all the aforementioned studies. Moreover, the relationship of SPARC overexpression with gastric-cancer progression is further confirmed by our study using GSEA and MTT assay.

The relationship between SPARC and chemotherapy drugs was another interesting topic. Anthracycline antibiotics are widely used in various chemotherapy regimens in combination with other drugs in GC. However, the efficacy is not entirely satisfactory due to drug resistance and no candidate predictor for drug sensitivity until now. The current results supported low expression of SPARC might predict Adr resistance in GC based on data derived from both GSEA and molecular experiment, and it was consistent with the findings in patients treated by Adr that higher SPARC expression predicted higher pathological complete remission rate in breast cancer and longer OS in diffuse large B-cell lymphoma, respectively [26, 27]. The potential mechanism was as follows: Adr-induced-apoptosis was enhanced by the activation of JNK [28], while Adr resistance was associated with the activation of AKT and the up-regulation of Notch1 and PTEN [29], respectively. Furthermore, SPARC was found to up-regulate the expression and activation of JNK [30], while suppress the activity of AKT and the signaling and expression of Notch1 [31–33], and the function of SPARC was negatively regulated by PTEN [34].

As for the relationship of SPARC and 5-FU, our preliminary results demonstrated that down regulation of SPARC in GC might enhance 5-FU sensitivity, which is distinct with the previous reports about SPARC in liver and colon cancers [35, 36], and the diversity is supposed to be related with the cancer type difference. Therefore, our future research would focus on the relationship of SPARC with 5-FU and other chemotherapy drugs in various cancers.

It was noticed that the percentage of male in our cohort was 75.2% (103/137), which seemed a little higher comparing with some other GC studies. However, as shown by the randomized clinical trials published in the recent 5 years, the ratio of male GC patients is between 64.6% and 73.9% (Supplementary Table S3). Then we randomly reviewed the high-quality retrospective studies for Chinese GC, and found the ratio of male GC patients to be about 57.8%–80.7% (Supplementary Table S3). Thus it was considered that there is no evidence for the impact of gender on the results of our study so far.

The heterogeneity of the current meta-analysis might be explained as follows. First, GC patients have different clinicopathological characteristics, such as age, race, TNM stage and the operation mode. In addition, only 2 of the 5 publications reported the patients' status of postoperative adjuvant therapy, and no information about the salvage therapy was elucidated. Second, the detection method for SPARC is different. Two of the studies used RT-PCR, whereas the other 3 used IHC. Moreover, the difference of primer sequence, antibody, and the cut off value selected might result in the different features of SPARC detection, such as the positive rate of 50%–72% and the histological location in stroma/cancer cells. Third, the HR data were got from different statistical approaches, such as the survival plots, the original reports or the calculation results. All the above factors might lead to between-study heterogeneity. However, it is difficult to perform meta-regression due to the small number of the included studies. Further multi-center researches using standardized methods are encouraged.

In summary, to evaluate the relationship between SPARC expression and the clinicopathological variables and the prognosis of GC, an IHC-based study of 137 cases and a meta-analysis based on published papers on PubMed were performed. Further survival analysis and GSEA with online data supported above results and revealed a potential association between SPARC expression and chemotherapy sensitivity in GC. The aforementioned several lines of investigation came to the conclusion that increased SPARC expression led to a worse clinical outcome. It is noticed that the evaluation standard and the immunostaining location of IHC for SPARC are not uniform, which directly hamper the integration of the existed literatures. Thus further larger-sample studies with standardized IHC staining/evaluation criteria for SPARC were warranted in future. Our future plan is to perform some more cellular experiments to study the association between SPARC expression and chemo-sensitivity in various cancers.

## MATERIALS AND METHODS

### Patients and tumor specimens in the IHC assay

Human specimens were approved to be used by the Ethics Committee of China Medical University (CMU). Clinical medical records and follow-up data of primary GC patients underwent initial surgical resection from May 2006 to Sep. 2008 were reviewed, and the ones with complete information and available specimens were recruited in the current study, while those undergone endoscopic mucosal resection, palliative resection, or preoperative chemotherapy were excluded. The OS was set on the period from the date of surgery to death or the most recent clinic visit (Sep. 2012).

Formalin-fixed, paraffin-embedded blocks were obtained from the archives of the Department of Pathology of the First Hospital of CMU, and three pathologists examined all specimens to confirm histopathological features. Tumors were staged according to AJCC criteria

### Tissue microarray and IHC

A tissue microarray was constructed in collaboration with Shanghai Biochip (Shanghai, China). Two punch cores of 1.0 mm were taken from the non-necrotic area of tumor foci or the corresponding non-tumor portion. IHC were performed as before [37] and the detailed protocol is shown in the Supplementary File S1. Negative control was obtained by the omission of the primary antibody in a slide with whole-tumor-section. All sections were evaluated blind by 2 experienced pathologists. If an inconsistency occurred, a third pathologist was consulted to achieve consensus. The staining intensity was scored as 0 (negative), 1 (weak), 2 (medium), and 3 (strong). Extent of staining was scored as 0 (0%), 1 (1 to 25%), 2 (26 to 50%), 3 (51 to 75%), and 4 (76 to 100%). The sum-indexes (−), (+), (++), and (+++) indicated final staining score of 0, 1–3, 4–5, and 6–7, respectively. For statistical analysis, sum-indexes (−) and (+) were defined as low SPARC expression, while sum-indexes (++) and (+++) were defined as high SPARC expression.

### Statistical analysis for IHC data and meta-analysis

SPARC expression was analyzed as a dichotomous variable (Low VS High). Gender (Female VS Male), Surgery D2 (Yes VS No), Location (Fundus & cardia VS Body VS Antrum & pylorus), Differentiation (Well & moderate VS Poor & mixed), Lauren type (Intestinal VS Diffused), T stage (T1-T3 VS T4), N stage (N0 VS N1-3), and M stage (M0 VS M1) were considered as categorical variables. Age was measured as a continuous variable. The associations between SPARC expression and the categorical variables were tested with Chi-square test or Fisher exact test, as appropriate. Welch's two-sample *t*-test was used to compute the *P* value for continuous variables.

Survival curves were plotted by the Kaplan-Meier (KM) method and compared with the log-rank test. HR and 95% CI was estimated using univariate and multivariate Cox PH models, respectively. Stepwise selection methods (including both “backward” and “forward” selection) were applied to construct the final multivariate model based on the Akaike information criterion (AIC) value. Internal validation of the final Cox model was checked with the estimation of covariable coefficients and 95% CIs using bootstrapping (1000 replications). The prediction accuracy of SPARC in the Cox model was assessed by the time-dependent receiver-operating characteristics (ROC) curves for the censored data. AUC was constructed according to Heagerty et al. [38], and the AUC (t) curve was plotted based on the evaluation of the risk scores to illustrate time-dependent sensitivity and specificity for the corresponding ROC curve at each observed event time.

A meta-analysis based on published literatures was used as an external validation. Using the methods described in the Supplementary File S1, clinicopathological variables including SPARC-detection method, SPARC positivity rate, HRs and the corresponding 95% CIs were collected, and the pooled HR of SPARC in GC was estimated.

Statistical analysis and meta-analysis were performed using R/meta software (R 3.0.2). All statistic tests in this study were two tailed with *P* < 0.05 as statistically significant, unless otherwise stated.

### Microarray, survival analysis, and GSEA

Totally 39 GEO datasets on PubMed were found to be about GC and GSE62254 is the only dataset with survival data available which was listed in the supplementary table of the relevant paper (PMID: 25894828) [39]. Series matrix data of 300 GC tissues from Korean patients using HGU133plus2 Affymetrix chip was downloaded. SPARC expression was trisected into 3 levels: low, median, and high. The cutoff was set to 0.33 and 0.67. Survival curves were plotted by the Kaplan-Meier (KM) method and compared with the log-rank test.

Gene Set Enrichment Analysis (GSEA) was performed using the software GSEA v2.2.2 (www.broadinstitute.org/gsea). SPARC expression level was dichotomized as low and high categories to annotate phenotype, and GC related gene sets from MSigDB was used [40–46]. All other parameters were set based on their default values.

### Cell culture, reagents and cellular experiments

The human GC cell line SGC7901 was obtained from the Academy of Military Medical Science (Beijing, China). The Adr -resistant variant of SGC7901 (SGC7901/Adr) was kindly provided by the Fourth Military Medical University (Xi'an, China). Antibodies for SPARC were obtained from Cell Signaling Technology (Beverly, MA, USA), and the secondary goat anti-rabbit and goat anti-mouse antibodies were purchased from Santa Cruz Biotechnology (Santa Cruz, CA, USA). Adriamycin sensitivity of SGC7901 with or without SPARC siRNA transfection was further investigated using MTT assay. The detailed information and the techniques for cell culture, Western Blot, siRNA, and MTT assay were described in our previous study elsewhere [20, 47] and the current Supplementary File S1.

## SUPPLEMENTARY MATERIALS




