# Supplementary material for: SPARC expression in gastric cancer predicts poor prognosis: Results from a clinical cohort, pooled analysis and GSEA assay
Source: Oncotarget. 2016 Sep 22;7(43):70211–22. doi: 10.18632/oncotarget.12191 (PMC5342547; doi:10.18632/oncotarget.12191)
Supplement: Supplementary file 1 [file oncotarget-07-70211-s001.pdf]

SPARC expression in gastric cancer predicts poor prognosis: results from a clinical cohort, pooled analysis and GSEA assay

Supplementary Materials

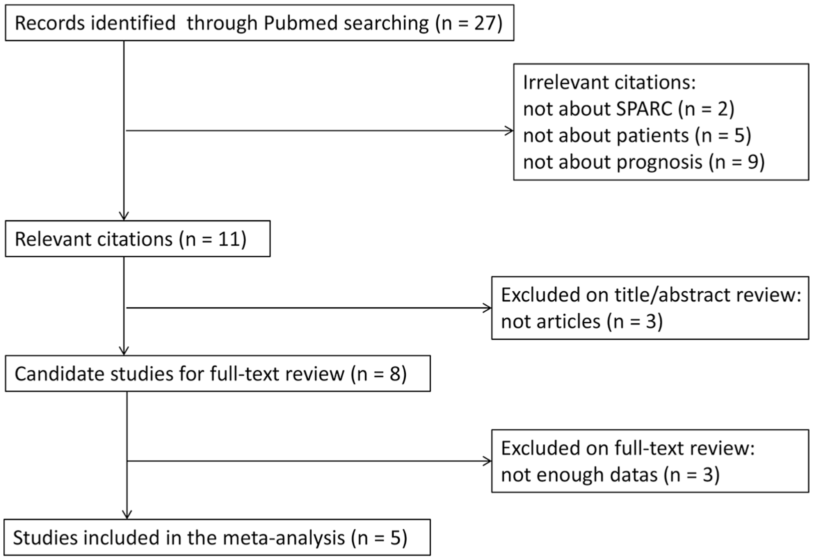

Supplementary Figure S1: PRISMA flow diagram for the inclusion and exclusion of searched papers.

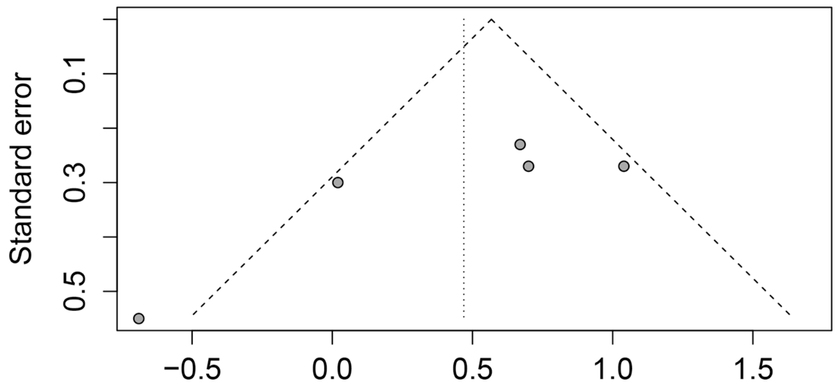

Supplementary Figure S2: Funnel plot showed that there was no asymmetry for the association between SPARC levels and overall survival.

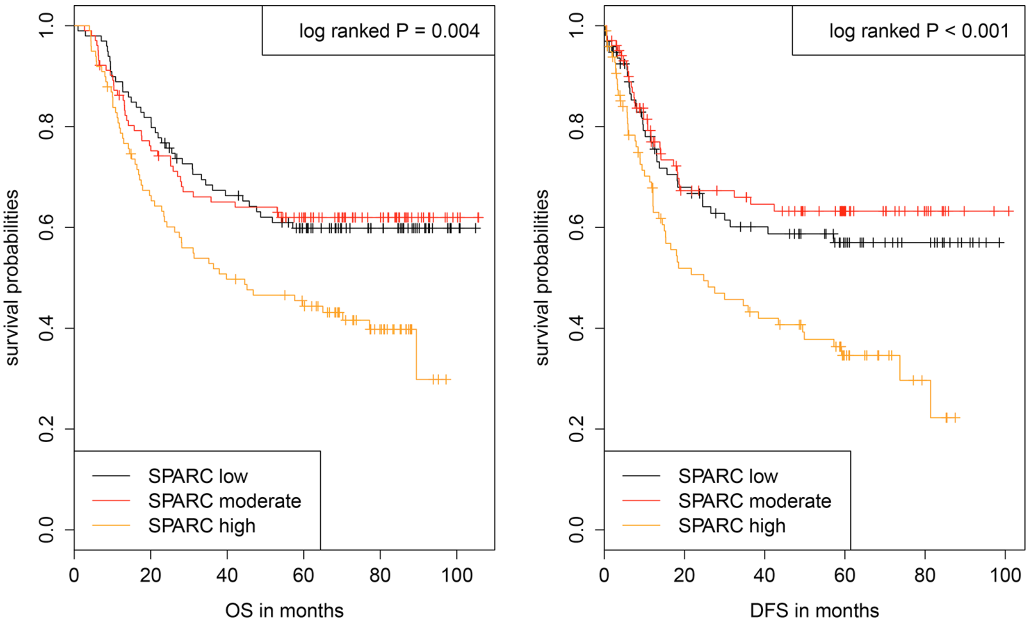

Supplementary Figure S3: KM survival curve and log-rank test for patients classified as showing either positive or negative SPARC expression in cancer. Patients with high SPARC expression exhibited a significant worse DFS and OS than those with low SPARC expression ($P = 0.004$ and $P < 0.001$, respectively; log-rank test).

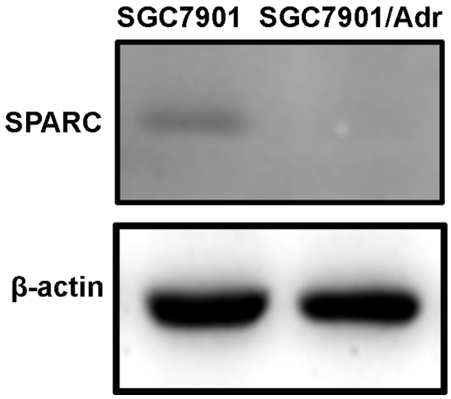

Supplementary Figure S4: Western blot analysis of SPARC in SGC7901/Adr and the parent cells. **SPARC expression was decreased in SGC7901/Adr cell line.**

Supplementary Table S1: Characteristics of studies for the overall survival and SPARC in gastric cancer. See Supplementary_Table_S1

Supplementary Table S2: Data of GSEA assay for the correlation of SPARC levels and gastric cancer related gene sets in MSigDB

Upregulated functional Gene Sets	ES	NES	NOM *p*-val	FWER *p*-val	SPARC Phenotype
VECCHI_GASTRIC_CANCER_ADVANCED_VS_EARLY_UP	0.872	2.853	0.000	0.000*	High Expression
KIM_LRRC3B_TARGETS	0.640	1.671	0.007	0.048*	High Expression
NOJIMA_SFRP2_TARGETS_UP	0.632	1.667	0.008	0.049*	High Expression
TIAN_BHLHA15_TARGETS	0.630	1.438	0.073	0.411	High Expression
TAKADA_GASTRIC_CANCER_COPY_NUMBER_DN	0.493	1.226	0.221	0.879	High Expression
KANG_DOXORUBICIN_RESISTANCE_DN	0.486	1.139	0.323	0.961	High Expression
NOJIMA_SFRP2_TARGETS_DN	0.414	1.051	0.417	0.992	High Expression
KANG_FLUOROURACIL_RESISTANCE_UP	0.425	1.043	0.419	0.995	High Expression
KANG_CISPLATIN_RESISTANCE_UP	0.336	0.771	0.748	1.000	High Expression
KIM_GASTRIC_CANCER_CHEMOSENSITIVITY	0.236	0.724	0.889	1.000	High Expression
VECCHI_GASTRIC_CANCER_ADVANCED_VS_EARLY_DN	−0.807	−3.436	0.000	0.000*	Low Expression
KANG_DOXORUBICIN_RESISTANCE_UP	−0.811	−2.856	0.000	0.000*	Low Expression
KANG_FLUOROURACIL_RESISTANCE_DN	−0.694	−1.886	0.000	0.003*	Low Expression

Supplementary Table S3: Percentage of male in previous literatures

Author	Journal	Year	Country	Male	Total	Percentage (%)
Ours				103	137	75.2
RCTs						
Jeeyun Lee	J Clin Oncol	2012	Korea	296	458	64.6
Tae Hyun Kim	Int J Radiat Oncol Biol Phys	2012	Korea	59	90	65.6
Hyung-Ho Kim	Ann Surg	2010	Korea	227	342	66.4
Hiroshi Imamura	Lancet Infect Dis	2012	Japan	240	355	67.6
Y-K Kang	Br J Cancer	2013	Korea	588	855	68.8
Changhoon Yoo	Ann Surg Oncol	2015	Korea	453	655	69.2
Mitsuru Sasako	J Clin Oncol	2011	Japan	720	1034	69.6
Aristotelis Bamias	Cancer Chemother Pharmacol	2010	Greece	100	143	69.9
Sung Hoon Noh	Lancet Oncol	2014	Korea	731	304	70.6
J. Kulig	Oncology	2010	Poland	211	295	71.5
Xiang Wang	Br J Cancer	2016	China	54	73	73.9
Retrospective						
Chao Yang	OncoTarget	2015	China	289	500	57.8
Kun Yang	OncoTarget	2016	China	956	1365	70.0
Daguang Wang	Int J Clin Exp Pathol	2015	China	449	634	70.8
Wei Zhao	OncoTarget	2016	China	428	600	71.3
Po Zhao	Int J Clin Exp Pathol	2015	China	153	192	79.7
H Q Xi	Br J Cancer	2014	China	259	318	80.7

Supplementary Table S4: Assessment of quality of studies

First author	Selection				Comparability	Outcome Assessment			Newcastle-Ottawa Scale
	1	2	3	4	5	6	7	8	
Tsutomu Sato	*	*	*	*	*	*	*	*	********
Konrad Franke	*	*	*	*	**	*	*	*	*********
Yong-Yin Gao	*	*	*	*	*	*	*	*	********
Hei-Cheul Jeung	*	*	*	*	**	*	*	*	*********
C-S Wang	*	*	*	*	*	*	*	*	********

Abbreviations: * = stars.

SUPPLEMENTARY MATERIALS

Immunohistochemistry of tissue samples

Immunohistochemical staining was performed by the antibody-linked dextran polymer method (EnVision; DAKO Cytomation). Deparaffinised and rehydrated 5-µm sections were incubated in 3% H2O2 for 10 minutes to block endogenous peroxidase activity. The sections were thereafter rinsed and incubated with 1:1000 SPARC antibody for 30 minutes. The second antibody-peroxidase-linked polymers were then applied, and the sections were incubated with a solution consisting of 20 mg of 3.3′-diaminobenzidine tetrahydrochloride, 65 mg of sodium azide, and 20 ml of 30% H2O2 in 100 ml of Tris-HCL (50 mM, PH7.6). After having been counterstained with Meyer's haematoxylin, the sections were observed under a light microscope.

META ANALYSIS

PubMed search strategy

Publications up to March 2016 were identified in the PubMed database (http://www.ncbi.nlm.nih.gov/pubmed/) using the following search strategy: ((((“gastric adenocarcinoma*”) OR “gastric cancer*”) OR Stomach Neoplasms [MeSH Terms])) AND ((((((“SPARC protein, human”[Supplementary Concept]) OR “Osteonectin”[Mesh]) OR Osteonectin [Title/Abstract]) OR BM40 [Title/Abstract])) OR SPARC [Title/Abstract]).

Inclusion criteria

Publications were included according to the following criteria: (1) studies should be published in English regardless of publication time; (2) reviews, comments, duplicated studies, animal or cell-line studies should be excluded; (3) gastric cancers should be removed by surgery, and pathologically confirmed; (4) SPARC expression should be detected by IHC or reverse transcriptase polymerase chain reaction (RT-PCR); (5) studies should evaluate the relationship between SPARC expression and clinicopathological variables or prognosis.

Quality evaluation of included literatures

Using the Newcastle-Ottawa Scale (NOS) standard, which is a system that evaluates literature quality with stars that max out at nine stars, we assessed the quality of the original studies in the meta-analysis. Studies were graded on an ordinal star scoring scale with higher scores representing studies of higher quality [1]. A study could be awarded a maximum of one star for each numbered item within the selection and exposure categories and a maximum of four stars could be given for the comparability of the two groups. The quality of each study was graded as either level 1 (0 to 5) or level 2 (6 to 9) [2]. As shown in Table 1, all the included studies are eligible for using in meta-analysis, according to the NOS standard.

Data extraction and analysis

With the eligible publications, we collected the following information: publication year, name of first author, the ethnicity and the number of patients, specimen type, technique for SPARC detection, and clinicopathological variables including cancer differentiation, Lauren type, and stage. If data from any of the above categories were not reported in the study, or were improper for the primary studies, items were treated as “N/A (not available)” or “unfit”. Additional or unreported information were not requested.

For ease of analysis, SPARC expression was categorized as high and low, and clinicopathological variables were combined into dichotomous categories as shown in Table S1. Hazard ratio (HR) and 95% confidence interval (CI) from univariate/multivariate analysis or data extrapolation were preferably taken. In those studies with only Kaplan-Meier (K-M) curve available, survival curves were read by Engauge Digitizer version 4.1 (downloaded from http://sourceforge.net), and HR, 95% CI, the significance as well as the orientation (favor protective or hazardous) were extracted from original publications as described by Tierney et al. [3].

Heterogeneity of publications was calculated with a Chi-square-based Q statistic and inconsistency index (I2) statistic ($P < 0.10$ and I2 > 50% indicated substantial heterogeneity). A random-effect model was used because

the heterogeneity was demonstrated. Log HRs were used to make the forest plot using R software, and 95% CI not overlap 0 was considered significant. Pooled HR and the 95% CI were obtained from log HR by calculation, and a HR > 1 (lnHR > 0) implied that SPARC high/positive expression predicted a worse survival of patients. To assess the stability of the results and the influence of our current study, we conducted cumulative meta-analysis. Egger's regression tests were performed to evaluate the publication bias.

Cell culture and western blot assay

The gastric cancer cell line SGC7901 and adriamycin-resistant variant of SGC7901 (SGC7901/Adr) were grown at 37°C and in 5% CO2. Cell was maintained in RPMI 1640 (Invitrogen, Grand Island, NY, USA) supplemented with 10% fetal calf serum (Invitrogen), 100 U/ml penicillin, and 100 mg/ml streptomycin. In the drug-resistant variant cell line SGC7901/Adr, the medium additionally contained 1 μg/ml Adr to maintain the drug resistance phenotype.

Preparing for western blot assay, the cells were washed twice with ice-cold PBS, lysed in 1% Triton lysis buffer on ice and quantified using the Lowry method. Proteins (40 μg) were separated using a 10% SDS-polyacrylamide gel and transferred electrophoretically onto polyvinylidene diÁuoride membranes (Millipore, Bedford, MA, USA). The membranes were blocked with 5% skim milk in Tris-buffered saline with Tween-20 for 1 h at room temperature, and subsequently incubated with primary antibodies targeting SPARC, β-actin, at 4°C overnight. After washing with TTBS, the membrane was reacted with the appropriate horseradish peroxidase–conjugated anti-rabbit (cat. no. sc-2491; 1:1,000) or mouse (cat. no. sc-2072; 1:1,000) secondary antibody (Santa Cruz Biotechnology, Inc.) for 30 min at room temperature. After extensive washing with TTBS buffer, the proteins were detected using the enhanced chemiluminescence reagent (SuperSignal Western Pico Chemiluminescent Substrate; Pierce, USA) and visualized with the Electrophoresis Gel Imaging Analysis System (DNR Bio-Imaging Systems, Israel).

Small interfering RNA transfections and cell viability assay

SPARC small interfering RNA (siRNA) and the control siRNA were obtained from Shanghai GeneChem Co., Ltd. (Shanghai, China). The siRNA sequences used were as follows: 5′-GCCACUUCUUUGCCACAAAT)-3′ (Forward) and 5′-TTTGTGGCAAAGAAGTGGC-3′ (reverse) for SPARC specific siRNA; and 5′-UUCUCCGAACGUGUCACGUTT-3′ (forward) and 5′-ACGUGACACGUUCGGAGAATT-3′ (reverse) for control siRNA. The gastric cancer cell line SGC7901 were transfected with the siRNAs using Lipofectamine 2000 (Invitrogen, Carlsbad, CA, USA) following the manufacturer's instructions. After 8 hours of siRNAs transfection, the cells were cultured with normal media for 24 hours. Then, cells were seeded into 96-well plates (2500 cells/well) and treated with Adriamycin at various concentrations (0.01, 0.1, 1, 10 and 100 μg/mL). After 24 h, MTT was performed to detect the cell viability. The calculative formula for suppression rate was as follows: cell suppression rate = $(1 - OD_{treated/ODcontrol}) \times 100$ %. The dose–response curve at different concentrations was charted to calculate the IC50 using a Probit regression model.

REFERENCES

1. Wells G, Shea B, O'connell D, Peterson J, Welch V, Losos M, Tugwell P. The Newcastle-Ottawa Scale (NOS) for assessing the quality of nonrandomised studies in meta-analyses. 3rd Symposium on Systematic Reviews: Beyond the Basics. 2000; 3–5.
2. Athanasiou T, Al-Ruzzeh S, Kumar P, Crossman M–C, Amrani M, Pepper JR, Del Stanbridge R, Casula R, Glenville B. Off-pump myocardial revascularization is associated with less incidence of stroke in elderly patients. The Annals of thoracic surgery. 2004; 77:745–753.
3. Tierney JF, Stewart LA, Ghersi D, Burdett S, Sydes MR. Practical methods for incorporating summary time-to-event data into meta-analysis. Trials. 2007; 8:16.
