# Supplementary material for: SPARC expression in gastric cancer predicts poor prognosis: Results from a clinical cohort, pooled analysis and GSEA assay
Source: Oncotarget. 2016 Sep 22;7(43):70211–22. doi: 10.18632/oncotarget.12191 (PMC5342547; doi:10.18632/oncotarget.12191)
Supplement: Supplementary file 2 [file oncotarget-07-70211-s002.docx]

Table S1: Characteristics of studies for the overall survival and SPARC in gastric cancer.

| Study | Race | Sample | Location | N | TNM stage | T stage | N stage | Lauren  (intestinal/diffuse) | Differentiation (good/poor) | Detection | Positivity rate (%) | HR extraction | HR | 95%CI |
| --- | --- | --- | --- | --- | --- | --- | --- | --- | --- | --- | --- | --- | --- | --- |
| Wang (2004) | Chinese | Frozen | Tissue | 43 | NM | 10/33^c^ | 11/32^e^ | 16/27 | NM | RT-PCR | 72.0 | DE | 2.84 | 1.01-7.95 |
| Franke (2009) | German | FFPE | Stromal | 152 | 36/35/39/42 | 15/67/60/10 | 45/52/36/19^g^ | 102/50 | NM | TMA-IHC | 68.4 | SC | 0.49 | 0.16-1.47 |
| Jeung (2011) | Korean | FFPE | Tissue | 66 | NM | NM | NM | NM | NM | WTS-IHC | 50.0 | Report-mul | 2.01 | 1.18-3.40 |
| Sato (2013) | Japanese | Frozen | Tissue | 227 | 30/60/72/65 | 121/106^a^ | 65/162^e^ | NM | 122/105 | RT-PCR | 50.0 | Report-uni | 1.96 | 1.25-3.08 |
| Gao (2015) | Chinese | FFPE | Cell | 78 | NM | 7/71^b^ | 20/58^d^ | 23/55 | 23/55 | WTS-IHC | 69.2 | SC | 1.02 | 0.56-1.85 |
| FFPE: Formalin-fixed, paraffin-embedded; NM: No mention; RT-PCR: reverse transcription-polymerase chain reaction; TMA: Tissue microarray; WTS: Whole tumor section; IHC: Immunohistochemistry; DE: Data extrapolation; HR: hazard ratio; SC: Survival curve; mul: multivariate; uni: univariate. ^a^: T1-2/T3-4; ^b^: T1-3/T4; ^c^: T1/T2-4; ^d^: N0/N1-3; ^e^: absent/present; ^f^: N0-x/N1/N2/N3 | | | | | | | | | | | | | | |
